# Successive Onset of Molecular, Cellular and Tissue-Specific Responses in Midgut Gland of *Littorina littorea* Exposed to Sub-Lethal Cadmium Concentrations

**DOI:** 10.3390/ijms18081815

**Published:** 2017-08-22

**Authors:** Denis Benito, Michael Niederwanger, Urtzi Izagirre, Reinhard Dallinger, Manu Soto

**Affiliations:** 1 CBET Research Group, Research Centre for Experimental Marine Biology and Biotechnology (PiE-UPV/EHU), University of the Basque Country UPV/EHU, Areatza Pasalekua, 48620 Plentzia-Bizkaia, Basque Country, Spain; denis.benito@ehu.eus (D.B.); urtzi.izagirre@ehu.eus (U.I.); 2 Institute of Zoology and Center of Molecular Biosciences Innsbruck (CMBI), University of Innsbruck, Technikerstraße 25, A-6020 Innsbruck, Austria; michael.niederwanger@uibk.ac.at

**Keywords:** *Littorina littorea*, metallothionein induction, midgut gland tubules, digestive cells, basophilic cells, connective tissue calcium cells, lysosomes, lipofuscin

## Abstract

Cadmium (Cd) is one of the most harmful metals, being toxic to most animal species, including marine invertebrates. Among marine gastropods, the periwinkle (*Littorina littorea*) in particular can accumulate high amounts of Cd in its midgut gland. In this organ, the metal can elicit extensive cytological and tissue-specific alterations that may reach, depending on the intensity of Cd exposure, from reversible lesions to pathological cellular disruptions. At the same time, *Littorina littorea* expresses a Cd-specific metallothionein (MT) that, due to its molecular features, expectedly exerts a protective function against the adverse intracellular effects of this metal. The aim of the present study was, therefore, to assess the time course of MT induction in the periwinkle’s midgut gland on the one hand, and cellular and tissue-specific alterations in the digestive organ complex (midgut gland and digestive tract) on the other, upon exposure to sub-lethal Cd concentrations (0.25 and 1 mg Cd/L) over 21 days. Depending on the Cd concentrations applied, the beginning of alterations of the assessed parameters followed distinct concentration-dependent and time-dependent patterns, where the timeframe for the onset of the different response reactions became narrower at higher Cd concentrations compared to lower exposure concentrations.

## 1. Introduction

Cadmium (Cd) is one of the most harmful metals, being considered as a cytotoxic [1], genotoxic [2,3] and carcinogenic agent [4]. It can provoke, moreover, radical stress [5,6] and exerts hormonal effects on eukaryotes [7,8]. Cd is also highly toxic to aquatic and marine animals [9,10,11] and is, therefore, of high relevance as a pollutant in the marine environment [12,13]. This is significant considering that, in spite of the normally rather low Cd concentrations in seawater [14,15], many marine species such as invertebrates and fish of upper trophic levels can accumulate high amounts of Cd in their tissues [16,17]. This may have important consequences for human seafood consumption [18,19], and may apply to heavily contaminated marine habitats to an even greater extent [20].

In particular, marine molluscs are able to accumulate metals from seawater reaching high tissue concentrations [21,22,23,24]. One of the marine organisms with an exceptionally high capability for Cd accumulation is the common periwinkle (*Littorina littorea*) [25,26]. This gastropod species lives on rocky shores of the intertidal zone of the North Atlantic Ocean, where it is intermittently exposed to adverse environmental conditions due to seasonal and daily fluctuations of water and oxygen supply, salinity, temperature, as well as the availability of mineral and metal ions [27,28]. *Littorina littorea* can resist these stressors by an adaptation of its metabolic pathways and its “cytoprotective repertoire” to the harsh conditions in its habitat [29,30]. In addition, *Littorina littorea* has apparently improved its fitness to survive by increasing its metal detoxification capacity [31,32].

Cd accumulation by *Littorina littorea* has been investigated from different approaches in order to determine the effects of sub-lethal exposure to this metal. Histopathological and histochemical analyses by means of autometallography and micro-analytical studies have been applied in order to better understand the toxicity mechanisms and the animal’s responses to Cd at different levels of biological complexity [12,32,33,34,35]. On the other hand, several studies have shown that, at the biochemical and molecular levels, an important response reaction of *Littorina littorea* to Cd exposure and other environmental stressors involves upregulation of metallothionein (MT) [31,36]. The metal binding features and tertiary structure of the MT of *Littorina littorea* have recently been elucidated and thoroughly reported [37,38].

As already indicated above, one of the central organs for Cd metabolism and Cd-induced response reactions in *Littorina littorea* is its midgut gland [32,34,39,40]. Generally, the molluscan midgut gland is a lobed organ composed of an interdigitating mass of blind-ending tubules. These tubules are bound together by connective tissue and muscle fibers and are irrigated by blood from the visceral sinus. In addition, gonad follicles are located in the connective tissue among the digestive tubules within the visceral mass of the midgut gland. The tubules converge to form major ducts which eventually drain into the stomach. The number of ducts may differ in a species-specific manner. Overall, at least two cell types are present in the tubules: digestive and basophilic cells. Their overall morphology is quite similar among all molluscan species [41,42]. It has previously been shown that the morphology, integrity and relative frequency of digestive and basophilic cells in the midgut gland of *Littorina littorea* might be subjected to significant alterations upon exposure to toxicants or environmental stressors [32,39,43,44,45,46]. Cd, in particular, can cause cytological alterations that impair the integrity and functionality of the midgut gland of littorinid gastropods [33]. These alterations include, for example, shifts and relocations of glycogen reserves [47], changes and re-structuring of lysosomal compartments, alterations of cell morphology and composition in midgut gland epithelia [39], as well as modifications in the volume density of the basophilic cells of the digestive tubules [32]. In fact, several of these cell-specific alterations have occasionally been applied as biomarkers for environmental pollution in marine habitats [32,48].

Altogether, Cd-induced cytological alterations in the midgut gland of *Littorina littorea* on the one hand, and molecular and biochemical response mechanisms such as increased MT mRNA concentrations on the other, must be considered as concomitant processes which complement each other. Up until now, however, we have lacked a clear perception regarding how these different response levels may be interconnected. The temporal succession of cellular and molecular response reactions may provide a first hint to this kind of interdependence. The working hypothesis of the present study was, therefore, the assumption that the onset of response mechanisms in the midgut gland of *Littorina littorea* provoked by sub-lethal Cd concentrations may be detected at very short but differing time-courses and may disappear after a given period of recovery.

Hence, individuals of *Littorina littorea* were exposed to two different Cd concentrations (“low” and “high”) at sub-lethal levels over a period of 21 days, followed by a recovery phase of 12 days. The general intention of our study was to follow and compare the time course of cellular and molecular response mechanisms to the Cd uptake in taken into the midgut gland of exposed snails, with a particular focus on the first 24 h of exposure.

## 2. Results

### 2.1. Cd Accumulation and Black Silver Deposits (BSD) in the Midgut Gland

**Figure 1:**
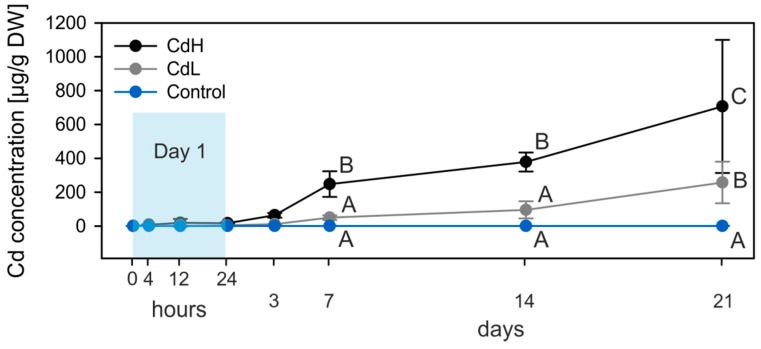
Cd accumulation in the digestive gland of the control (blue lines and symbols) and Cd-exposed winkles (grey line and symbols: CdL = 0.25 mg Cd /L; black line and symbols: CdH = 1 mg Cd /L). Mean values and standard deviations (*n* = 4) are shown. The significance of the accumulation curves (CdL and CdH) was confirmed by ANOVA (*p* ≤ 0.001). The significant differences of values between Cd-exposed and control individuals at different time points (Holm–Sidak pairwise multiple comparison, significance level 0.05) are indicated by different letters. Sampling points during the first 24 h (Day 1) are under-laid in blue.

Cd accumulation in the midgut gland of metal-exposed winkles (CdL “Cd Low”, 0.25 mg Cd/L and CdH “Cd High”, 1 mg Cd/L) was delayed in time, with significant differences from control values appearing at day 7 for CdH winkles, and for both metal treatment groups at the end of exposure (Figure 1).

**Figure 2:**
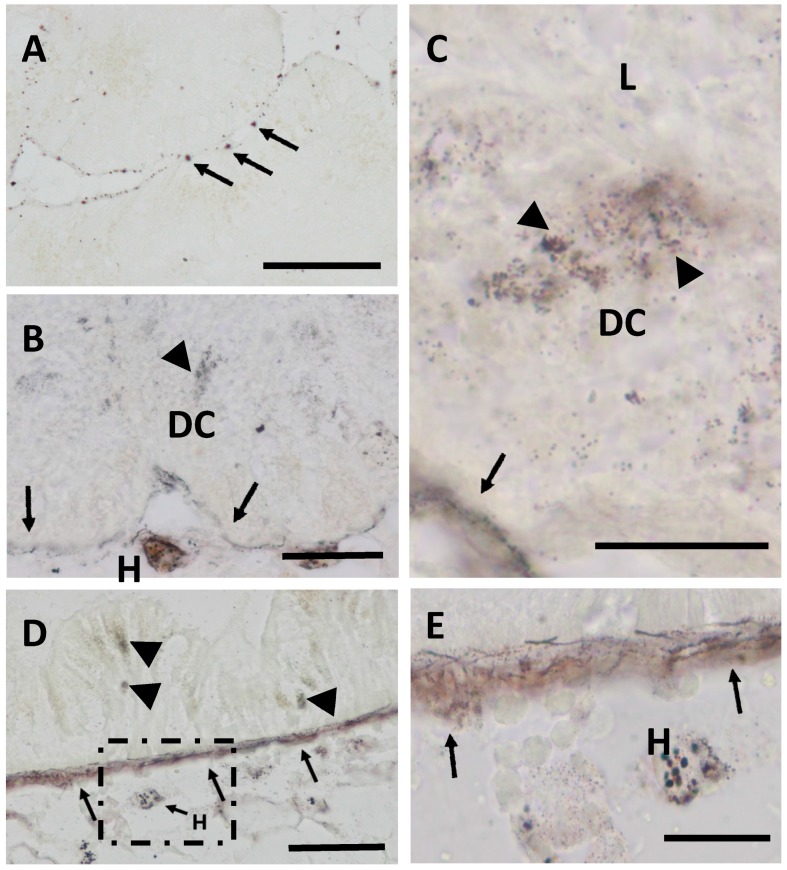
Autometallography staining of the midgut gland of winkles. (**A**) Controls, black silver deposits (BSD) in the basal lamina (arrows) of midgut gland tubuli (scale bar: 100 µm); (**B**) CdH (21 days), BSD in digestive cell lysosomes (arrowheads) and in the basal lamina (arrows) of midgut gland tubules. Note the presence of BSD within hemocytes (H) of the interstitial connective tissue (scale bar: 50 µm); (**C**) CdH (21 days), detail of BSD in digestive cell (DC) lysosomes (arrowheads) near the tubular lumen (L) and in the basal lamina (arrows) of a midgut gland tubule (scale bar: 20 µm); (**D**) CdH (21 days), BSD in the basal lamina of the epithelium of the stomach (arrows), in the lysosomes of epithelial cells (arrowheads) and in hemocytes (H) of the connective tissue (scale bar: 50 µm); (**E**) CdH (21days), inset of figure (**D**) (square), showing in detail BSD in hemocytes and in the basal lamina of the stomach epithelium (arrows).

Concomitantly, metal detection by autometallography revealed only scarce black silver deposits (BSD) in the digestive cells of control winkles. Instead, they were mainly localized in the basal lamina (histological sense) of the digestive tubules (Figure 2A). After Cd exposure, BSD became more conspicuous, being evident in the basal lamina of the tubular epithelium and in lysosomes of tubular digestive cells, as well as in hemocytes of the interstitial connective tissue (Figure 2B–E). Moreover, BSD were also detected in the basal lamina of the adjacent stomach epithelium and in lysosomes of the stomach enterocytes (Figure 2D,E). During the period of recovery, the amount of BSD in the connective tissue cells of the midgut gland and in the digestive epithelia of the stomach declined again.

### 2.2. Increase of Metallothionein mRNA Concentration

**Figure 3:**
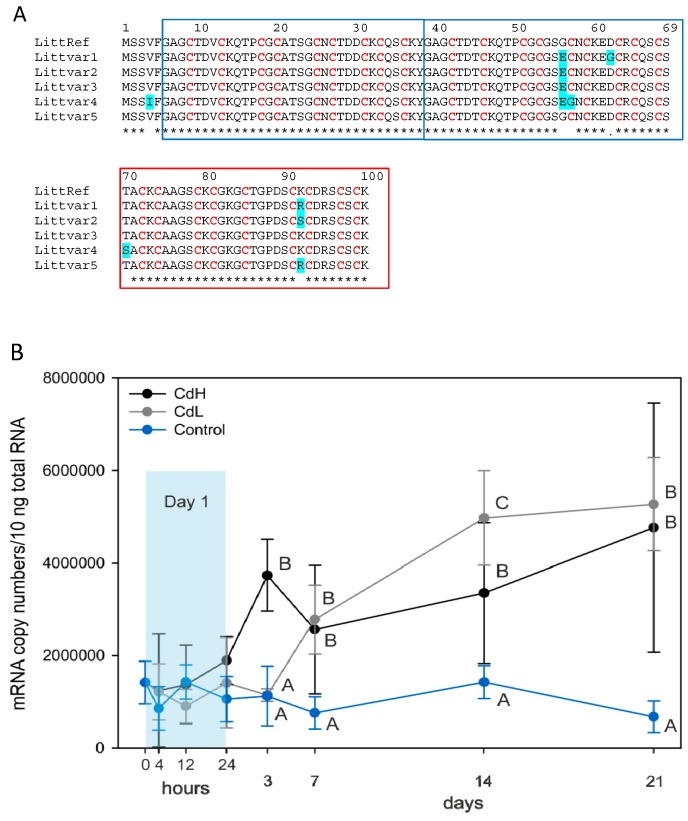
(**A**) Translated protein sequences of allelic variants of *Littorina littorea*, all of them caused by single nucleotide polymorphisms (SNPs) in the coding region of the respective gene. The sequence variants (Littvar1, Littvar2, Littvar3, Littvar4, Littvar5) are aligned with the first reported reference sequence (LittRef) of the *Littorina littorea* MT (GenBank Acc.Nr.: AY034179.1) [31]. Exchanged amino acid residues in the allelic variants are marked in blue. The tri-partite structure of the protein into different domains is indicated by blue (α domains) and red frames (β domain) [37]. The allelic variant sequences are available from GenBank under the acc. nrs.: KY963497 (Littvar 1), KY963498 (Littvar2), KY963499 (Littvar3), KY963500 (Littvar4) and KY963501 (Littvar5); (**B**) mRNA transcription of the Cd metallothionein (MT) gene of *Littorina littorea* in control animals (Control, blue line and symbols) and in winkles exposed to nominal Cd concentrations of 0.25 mg/L (CdL, grey line and symbols) and 1.00 mg/L (CdH, black line and symbols) over a period of 21 days. Mean values and standard deviations (*n* = 4) are shown. The significance of the mRNA transcription curves was confirmed by ANOVA (*p* ≤ 0.001). The significant differences of values between Cd-exposed and control individuals at different time points (Holm–Sidak pairwise multiple comparison, significance level 0.05) are indicated by different letters (A, B, C). Sampling points during the first 24 h (Day 1) are under-laid in blue.

Until this point, we had only found one species of metallothionein (MT) mRNA in the midgut gland of *Littorina littorea*. However, there was a conspicuous allelic variability across individuals, with at least five different allelic MT variants, as seen in Figure 3A. Hence, the respective mRNAs and the translated amino acid sequences of the MT coding regions proved to be not completely identical to a previously published (reference) sequence from a study focusing on MT expression in periwinkles exposed to environmental stressors [31] (Figure 3A). For quantitative real-time PCR, the reference MT mRNA sequence was applied.

MT mRNA copy numbers in the midgut gland of control and Cd-exposed winkles are shown in Figure 3B. It appeared that the response of MT gene transcription was somewhat delayed with respect to the start of metal exposure, with a first significant peak of mRNA concentration at day 3 for the CdH group and at day 7 for the CdL group (Figure 3B). Moreover, while the intensity of Cd exposure (CdL or CdH) had an influence on the response time of the increase of MT mRNA concentration, the upper level of MT mRNA transcription reached during the experimental time course remained the same for both Cd exposure groups (CdL and CdH) (Figure 3B).

**Figure 4:**
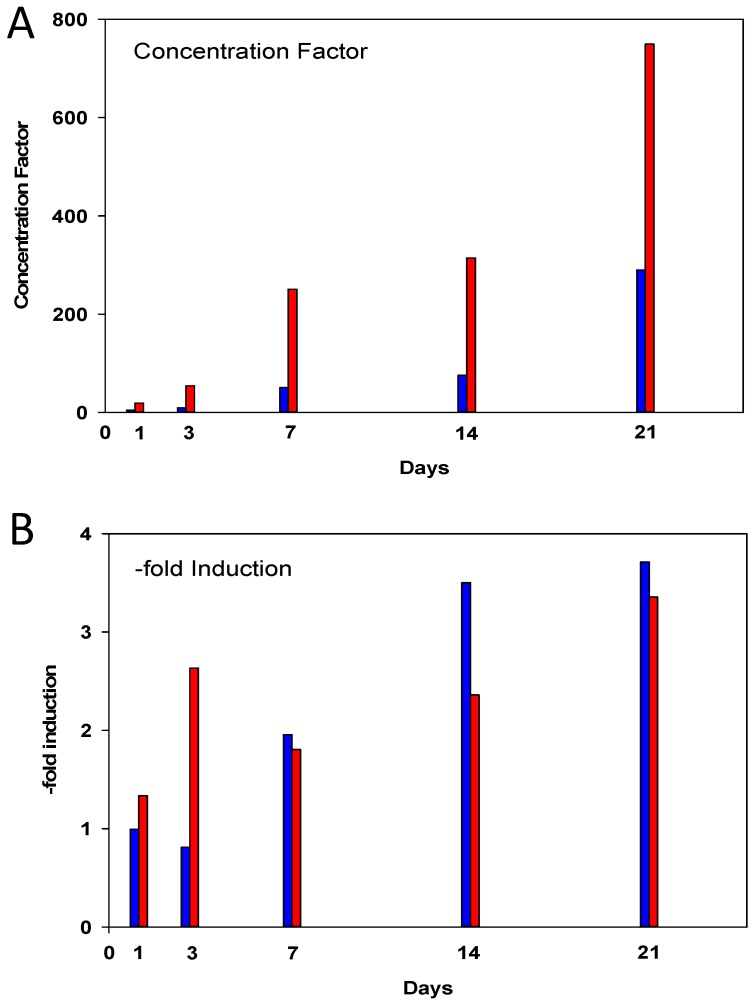
(**A**) Concentration factor for Cd in the midgut gland of *Littorina littorea*. Blue bars represent the low Cd exposure group (CdL), the red bars represent the high Cd exposure group (CdH). All values refer to respective control concentrations at the beginning of the experiment; (**B**) Fold induction factor for the increase of the MT mRNA concentration. Blue bars represent the low Cd exposure group (CdL). Red bars represent the high Cd exposure group (CdH). All values refer to the respective control mRNA copy numbers at the beginning of the experiment (set to 1).

In fact, Cd concentration factors in the midgut gland of metal-exposed winkles increased until the end of exposure to about 250 times in the CdL group and to about 750 times in the CdH group (Figure 4A). At the same time, the increase in MT mRNA concentration rose from an initial value of about 1.7-fold to approximately 7.5-fold in both groups of metal-exposed winkles (Figure 4B), irrespective of whether exposed individuals had enriched the Cd in their midgut glands with lower (CdL) or much higher (CdH) concentration factors.

### 2.3. Cd-Induced Response Patterns at the Cellular and Tissue Levels

#### 2.3.1. Digestive Cells

**Figure 5:**
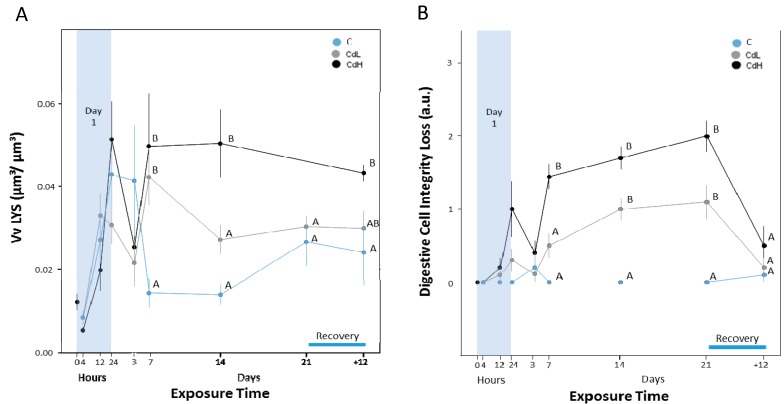
(**A**) Progression of volume density of lysosomes (Vv_LYS_) in the digestive cells of midgut gland tubules of *Littorina littorea*. Means (symbols) and standard errors (bars) are shown. Different letter codes between single values of the same time point indicate statistically significant differences (*p* < 0.05); (**B**) Course of integrity loss of digestive cells (DCI) in the midgut gland tubular epithelium, expressed in arbitrary units (a.u.) (see Table 1 in the Materials and Methods section for explanation). Means (symbols) and standard errors (bars) are shown. Different letter codes between single values of the same time point indicate statistically significant differences (*p* < 0.05).

The volume density of digestive cell lysosomes (Vv_LYS_) in the midgut gland oscillated for all treatment groups tover the first three days of exposure (Figure 5A). Significant differences between Cd-exposed and control snails were observed after 7 days of exposure. From that point on, the V_VLYS_ values of winkles exposed to the higher Cd concentration (CdH)—but not those of the CdL group—remained significantly elevated over the whole period of exposure, without regeneration during the recovery period (Figure 5A). Upon microscopic examination, digestive cell lysosomes in the midgut gland of the CdH group appeared to be larger and aggregated in bigger clusters compared to the situation in the CdL and control groups, where lysosomes seemed to have a bigger diameter and to be aggregated in smaller groups, or did not appear in clusters at all. As shown by autometallography, the digestive cell lysososmes from Cd-exposed winkles contained dense signals of BSD, indicating metal loads (Figure 2B,C).

The digestive cell integrity in the midgut gland tubules of the control group remained stable over the entire the exposure period (Figure 5B). In contrast, the loss of digestive cell integrity increased significantly in Cd-exposed snails (CdL and CdH) from day 7 until day 21 of exposure, but returned to control levels during the recovery period (Figure 5B).

**Figure 6:**
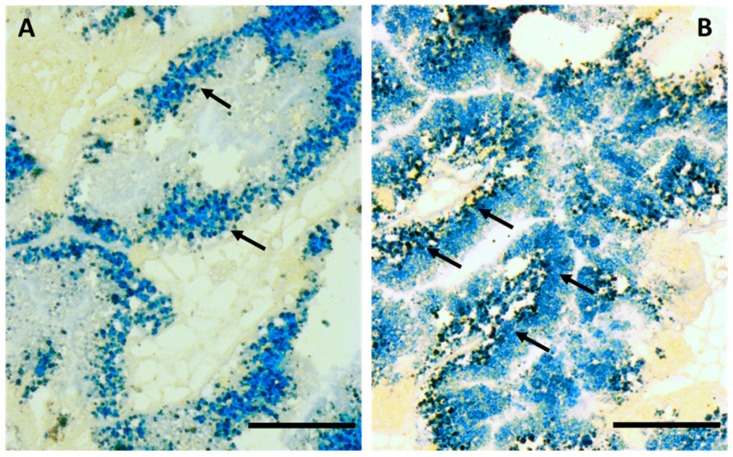
Lipofuscin staining in digestive cells of the tubular epithelium in the midgut gland of (**A**) a control periwinkle and; (**B**) an individual of the CdH group after 21 days of exposure. Scale bars: 200 µm; arrows indicate lipofuscin granules.

The presence of lipofuscin granules in digestive cells of the midgut gland tubular epithelium (Figure 6) increased gradually with increasing exposure time and Cd concentrations. Overall, lipofuscin was more abundant in the CdH group than in the CdL group. No changes were found during the experiment in the control group, in which the amount and intensity of lipofuscin staining remained stable.

#### 2.3.2. Basophilic Cells

**Figure 7:**
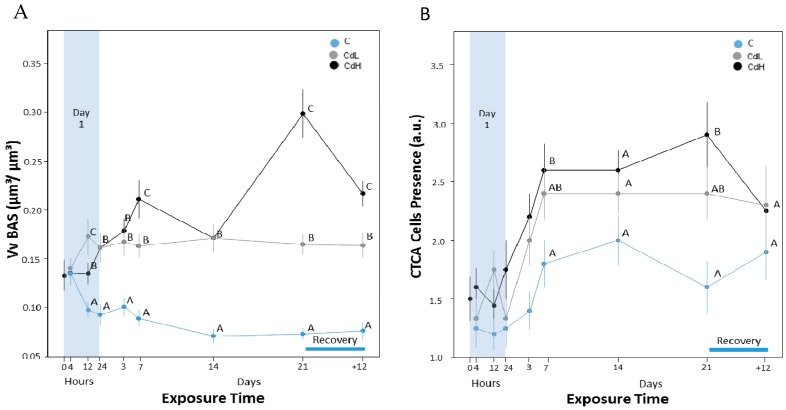
(**A**) Time course of volume density of basophilic cells (V_VBAS_) in the midgut gland tubules of *Littorina littorea*. Means (symbols) and standard errors (bars) are shown. Different letter codes between single values of the same time point indicate statistically significant differences (*p* < 0.05); (**B**) Progression of the presence of connective tissue calcium cells (CTCA) in the midgut gland of periwinkles, expressed in arbitrary units (a.u.) (see Table 1 in the Materials and Methods section for explanation). Means (symbols) and standard errors (bars) are shown. Different letter codes between single values of the same time point indicate statistically significant differences (*p* < 0.05).

**Figure 8:**
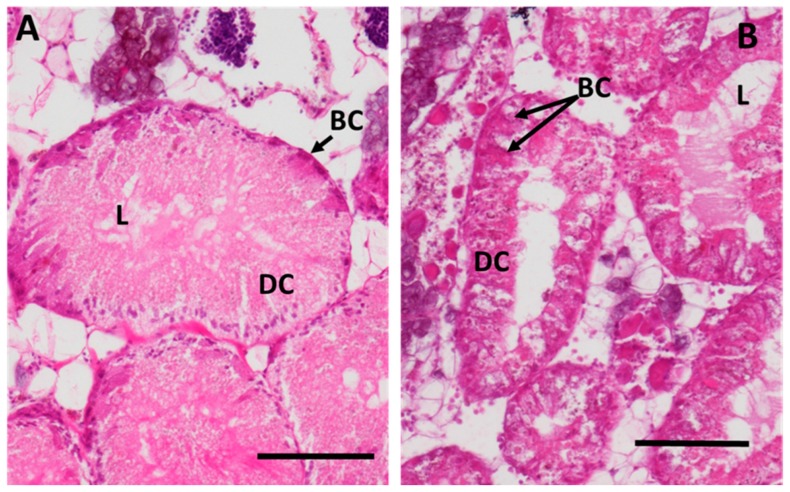
(**A**) Detailed view of the midgut gland tubules (hematoxylin-eosin staining) of a control periwinkle and (**B**) of a periwinkle of the CdH group after an exposure of 21 days. Note the drastic increase of the relative number of basophilic cells (arrows) and the enlarged lumen in (**B**), compared to control conditions (**A**). Scale bars: 200 µm; Abbreviations: L: lumen, DC: digestive cells, BC: basophilic cells.

Apart from a slight initial decrease, there was no significant change in the relative proportion of basophilic and digestive cells in the midgut gland tubuli of control periwinkles, expressed as the volume density of basophilic cells (V_VBAS_). In contrast, both Cd concentrations (CdL and CdH) provoked a significant increase in the volume density of basophilic cells (V_VBAS_) in metal-treated animals after only 12 h of exposure (Figure 7A). These values increased further until day 21 of exposure in both groups (CdL and CdH), leading to a clearly visible difference in the histological appearance (Figure 8) between the control (Figure 8A) and Cd-exposed individuals (Figure 8B).

During the period of recovery, the V_VBAS_ values in exposed groups (CdL and CdH) did not achieve the values of the control group any more, even though the value of the CdH group decreased slightly from day 21 of exposure till the end of the recovery period (Figure 7A). 

The integrity of the basophilic cells showed the same trend for all treatment groups, as observed in the digestive cells (data not shown).

#### 2.3.3. Connective Tissue Calcium Cells

**Figure 9:**
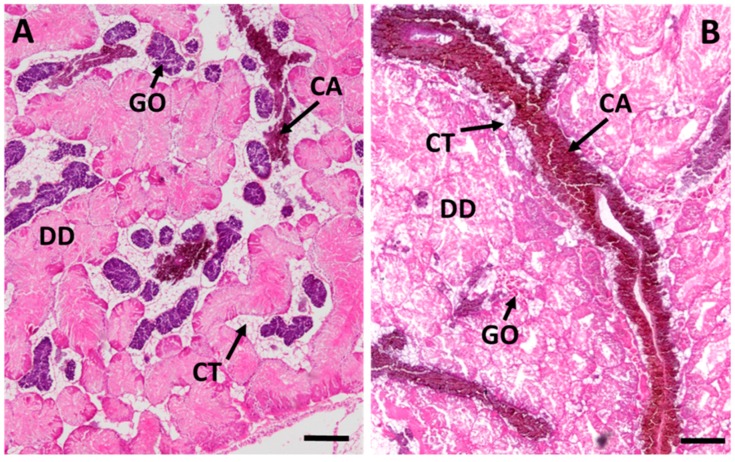
Overall view of the midgut gland/gonad complex stained with hematoxylin-eosin. (**A**) control periwinkle (male); (**B**) a periwinkle of the CdH group after 21 days of exposure (female). Scale bars: 200 µm; Abbreviations: DD: digestive tubular diverticula, CA: Connective Tissue Calcium cells, CT: connective tissue; GO: gonad tissue.

The presence of connective tissue calcium cells in the midgut gland (Figure 7B) increased for all groups during the experiment, although this increase was higher in the Cd-exposed animals (CdL and CdH) than in the controls (Figure 7B). However, only values of the CdH group were significantly different from the control values after 7 and 21 days of metal exposure. After the period of recovery, values decreased in both Cd exposure groups (CdL and CdH), reaching control values again (Figure 7B). A microscopic inspection of histological midgut gland sections confirmed the increased presence of connective tissue calcium cells in Cd-exposed winkles (Figure 9).

#### 2.3.4. Midgut Gland Structure and Integrity

**Figure 10:**
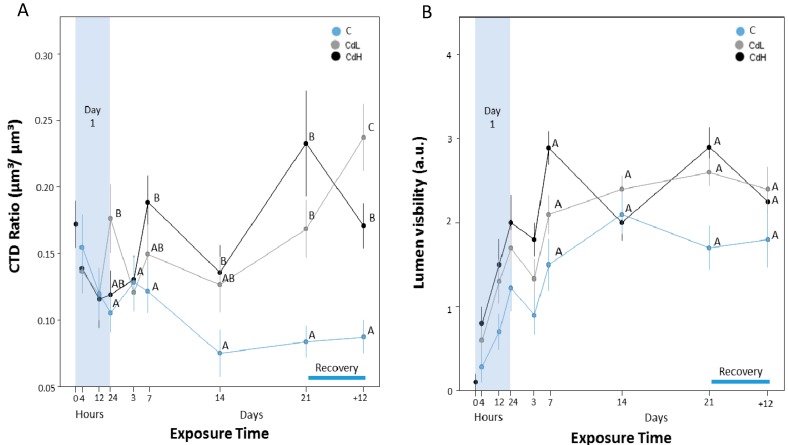
(**A**) Progression of structural integrity in the midgut gland/gonad complex of *Littorina littorea*, expressed by the connective tissue to digestive tubule ratio (CTDr). Means (symbols) and standard errors (bars) are shown. Different letter codes between single values of the same time point indicate statistically significant differences (*p* < 0.05); (**B**) Progression of lumen visibility of midgut gland tubules, expressed in arbitrary units (a.u.) (see Table 1 in the Materials and Methods section for explanation). Means (symbols) and standard errors (bars) are shown. Different letter codes between single values of the same time point indicate statistically significant differences (*p* < 0.05).

As shown in Figure 10 by quantitative parameters, the structural integrity in the midgut gland/gonad complex was subjected to apparent alterations over the course of the experiment. For example, the connective tissue to digestive tubular ratio (CTD) increased over the exposure time in the midgut gland of Cd-treated snails (CdL and CdH) (Figure 10A). Statistically significant differences between the Cd-exposed groups and the control group appeared from the seventh day on. During the recovery period, no return to control levels was noticed (Figure 10A). Concomitantly, the visibility of the tubular lumen in the midgut gland increased in all treatment groups during the experiment, including the control group (Figure 10B), although no statistically significant difference could be assessed between different treatments. Nevertheless, values were consistently higher in the Cd-exposed groups (CdL and CdH), and especially in the CdH group.

Upon microscopic observation of histological sections, the lumen visibility in the midgut gland of Cd-exposed winkles was evident when compared to control individuals (Figure 8). Moreover, the reduction of the digestive gland integrity was clearly visible, especially when compared with the digestive gland structure of control individuals (Figure 9).

#### 2.3.5. Integrity of Digestive Tract Epithelium

**Figure 11:**
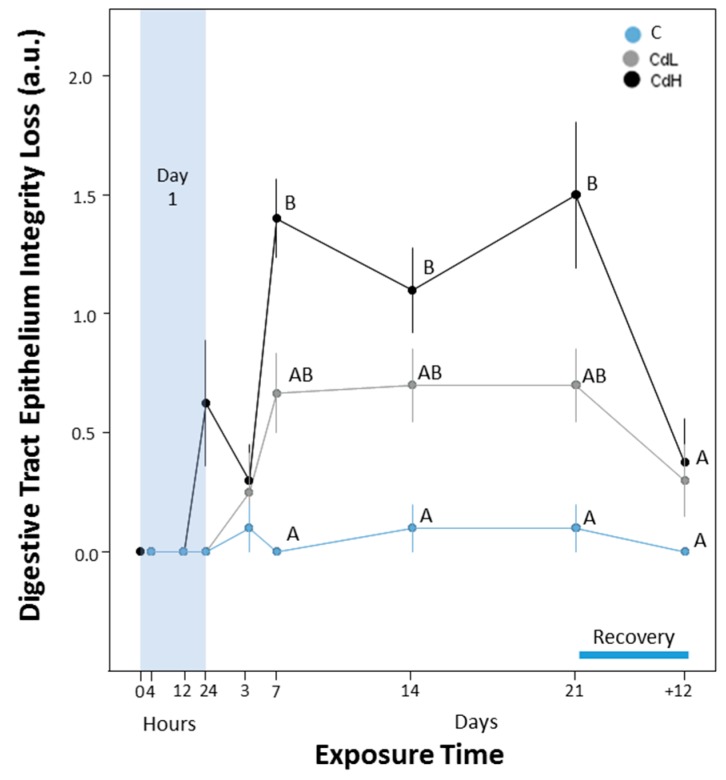
Course of the integrity loss of the epithelia of digestive tract (DTI), expressed in arbitrary units (a.u.) (see Table 1 in the Materials and Methods section for explanation). Means (symbols) and standard errors (bars) are shown. Different letter codes between single values of the same time point indicate statistically significant differences (*p* < 0.05).

**Figure 12:**
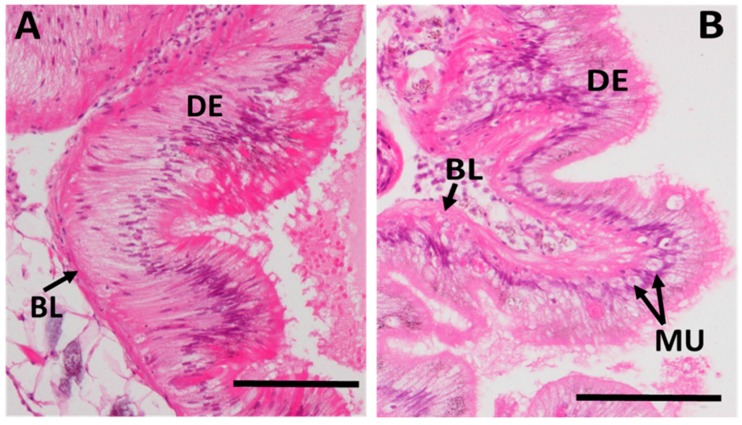
Sections of the stomach (digestive tract) stained with hematoxylin-eosin. (**A**) Control periwinkle; (**B**) Periwinkle of the CdH group after 21 days of exposure. Note the loss of integrity in the stomach epithelium (arrows) and the increase of mucocytes as a result of Cd-exposure. Scale bars: 200 µm; Abbreviations: DE: Digestive epithelia, BL: Basal lamina, MU: Mucocytes.

Apart from the midgut gland, a loss of tissue integrity due to Cd exposure was also observed in the digestive tract. When measured quantitatively, the structural integrity loss of epithelial cells of the digestive tract was pronounced, especially in the CdH-exposed winkles, when compared to control individuals (Figure 11). This was confirmed upon inspection of the digestive tract tissue condition in histological sections (Figure 12). Apart from a slight disintegration of the tissue structure, the digestive tract epithelium of Cd-exposed individuals also contained an increased density of mucocytes due to metal exposure (Figure 12A) compared to the intact structure of control winkles (Figure 12B).

## 3. Discussion

### 3.1. Cellular Cadmium Accumulation and the Significance of MT mRNA Increase

The Cd accumulation capacity of *Littorina littorea* is huge, considering the rather low metal concentrations of the exposure medium in the present study, with nominal Cd concentrations of 0.25 mg/L (effective: 0.24 mg/L) in the CdL exposure and of 1.0 mg/L (effective: 0.85 mg/L) in the CdH exposure. Both concentrations are far below the known LC_50_ (96h) values for Cd in *Littorina littorea* [49,50], and are even slightly lower than sub-lethal Cd concentrations applied in some previous studies with this species [39,47]. In spite of this, one has to consider that real Cd concentrations are, even in seawater samples from contaminated habitats, about 1000 to 10,000 times lower than the experimental concentrations applied in the present study [14]. All the more remarkable is the resistance of *Littorina littorea* in its various response patterns to accumulated midgut gland Cd concentrations of more than 250 µg/g dry wt. in the low exposure group (CdL) and about 700 µg/g dry wt. in the high exposure group (CdH) (Figure 1). These concentrations are about 290 times (CdL) and up to 750 times (CdH), respectively, higher than the control levels detected in the midgut gland tissue of uncontaminated winkles (Figure 4A).

Evidently, a considerable proportion of the accumulated Cd is retained by cellular compartments. This is suggested by the results of autometallography (Figure 2) which shows a distinct increase of BSD spots in the basal lamina and digestive cells of midgut gland tubules, and in the connective tissue of the midgut gland/gonad complex. Cd exposure also led to marked BSD signals in the basal lamina of the stomach, in the lysosomes of the stomach epithelial cells, and in hemocytes of the connective tissue near the basal lamina of the stomach. Autometallography as a histological method was developed for the visualization of metal sulphide and selenide compounds in cellular structures [51]. A number of more recent studies have demonstrated that this method works excellently for the localization of metal deposits in metal-exposed marine molluscs [35], including *Littorina littorea* [32,52]. Autometallography fails, however, to demonstrate the presence of metal ions bound to cytosolic MT molecules [24], where metal ions such as Cd^2+^ are coordinated by the sulphur atoms of the protein’s cysteine residues [53,54]. Only when these stable Cd-metallothionein complexes are semi-digested in the digestive cell lysosomes, exposing the bound Cd ions, or when these complexes are sufficiently concentrated in lysosomes, can autometallographed black silver deposits be formed to reveal the toxic metal [52]. Therefore, lysosomes can contain degradation products of MTs and serve as a final storage site of degraded MTs and, possibly, of other metal-binding proteins. In any case, the speciation of Cd is the same (Cd^+2^) in the cytosol and in the lysosomal pool.

Yet, the participation of MT in the process of Cd accumulation and detoxification by *Littorina littorea* is essential [36,55], as also demonstrated by the present study. The increase of MT mRNA concentration in Cd-exposed periwinkles can be considered, therefore, as an efficient protective molecular response to the uptake intake of a metal ion that is highly toxic to most animal species at concentrations far below those observed in *Littorina littorea*. In fact, the MT protein of *Littorina littorea* possesses highly Cd-selective binding features [38]. Its Cd-binding capacity has been optimized through evolution by the addition of a third Cd-binding protein domain. The observed sequence variability caused by single nucleotide polymorphisms (SNPs) mostly affects non-critical amino acid positions (Figure 3A). Hence, it can be assumed that the overall performance of the MT as a cytosolic Cd complexing agent is not significantly affected. The tertiary structure of this protein has recently been elucidated by means of solution NMR, making the MT of *Littorina littorea* the first MT so far to have been shown to have a proven three-domain architecture [37].

Interestingly, the induction factors for the increase in MT mRNA concentration detected in the midgut gland of Cd-exposed winkles are, at about 6.6-fold, rather moderate (see Figure 4B) compared to those observed for MT genes in other marine species [56,57,58]. Moreover, it appeared that the upper limits of the increase of MT mRNA concentration in Cd-exposed winkles increased moderately with exposure time, but almost without significant differences between CdL and CdH-exposed individuals throughout (Figure 2B). This is also reflected by the pattern of increased MT induction values (Figure 4B). Instead, the rise of the MT mRNA concentration started faster in CdH than in CdL-exposed snails (Figure 2B). In the CdH group, the first significantly elevated values of MT mRNA concentration were observed after three days of metal exposure. This is a slightly delayed reaction compared with the early response of some cytological alterations, especially in the volume density of basophilic cells (V_VBAS_), which was assessed only a few hours after the start of Cd exposure (Figure 7A). An obvious interpretation for this may be that, due to its high selectivity and capacity for Cd binding, the MT of *Littorina littorea* may already efficiently work at only moderate expression rates, maintaining its stress response capability in this way in an energy-saving mode [37]. In fact, the minimization of energy expenditure in metal-stressed invertebrates is often achieved by metabolic depression or other energy-saving strategies [59,60]. In such situations, the energetic demand for the upregulation of cellular protective mechanisms such as MT induction is preferably kept as low as possible [61]. The Cd-specific binding character and the extended loading capacity for additional Cd^2+^ ions due to the three-domain structure of the *Littorina littorea* MT may comply with these energy-sparing requirements, conferring to periwinkles an evolutionary advantage in coping with Cd stress [37]. This is all the more significant if we consider that an increase in MT mRNA concentration in *Littorina littorea* may also occur in response to environmental, non-metallic stressors [31].

Overall, the near concomitance of an increasing MT mRNA concentration with enormous alterations at the cellular level (see below) reinforces the impression that increased MT mRNA levels in the midgut gland of periwinkles apparently serves the purpose of protecting *Littorina littorea* from potentially adverse Cd effects. This interpretation is consistent with the view that, as well as in many other animal species, MTs may exert a protective role against the adverse effects of Cd^2+^ [62]. For *Littorina littorea*, this may be especially important during periods of increased Cd exposure via the external medium (as in the present study). The protective role of MT may also be important, however, under any condition that leads to significant cytological re-structuration or cellular wear in the midgut gland of winkles, with an increased potential for metal ions to leak out from their intracellular compartments [43]. This may occur due to the impact of non-metallic contaminants [44,63,64,65], the influence of fluctuating environmental stressors [31,66], or because of seasonal alterations of the physiological state of periwinkles [45,67].

### 3.2. Cadmium-Induced Cellular Response Patterns in the Midgut Gland

#### 3.2.1. Digestive Cells

Cd exposure led to alterations in the shape and integrity of digestive cells in midgut gland tubules (Figure 5B). One of the reasons for this is that Cd-stressed digestive cells cast off their apical parts and become flatter [33]. In addition, the present study also shows that the relative number of digestive cells declined in favor of an increase of the volume density of basophilic cells (V_VBAS_) (see Figure 7A). This seems to be in contrast with previous findings, where it was shown that Cd exposure of *Littorina littorea* stimulates digestive cell proliferation [32]. At the same time, however, Cd also causes an increase of digestive cell mortality. Overall, their number actually decreases in relation to basophilic cells, due to the fact that they die at a faster rate than they proliferate, as also observed in previous experiments [32]. In the present study, these alterations caused a drastic change of the midgut gland tubular morphology, with an increased visibility of the tubular lumen and a thinner surrounding epithelium consisting predominantly of basophilic cells (Figure 8 and Figure 10). Generally, Cd is known for its stimulatory effects on cell proliferation in mammals [68], and even so in molluscs, including terrestrial [69,70] and marine gastropods like *Littorina littorea*, especially under the influence of pollutant stressors [32,39]. Occasionally, cell proliferation in gastropods has been observed to be accompanied by programmed cell death [70,71,72]. Both phenomena indicate that gastropod tissues can be subjected to extensive structural alterations in response to environmental stressors, documenting the huge plasticity of these animals in adapting to the fluctuating and sometimes adverse conditions of their environment [67,73].

Under Cd exposure, in particular, digestive cells assume another important metabolic task: their lysosomal system is apparently involved in Cd storage and detoxification. This is clearly demonstrated in our study by the increase of autometallographic BSD signals in lysosomes of the midgut gland digestive cells of Cd-exposed winkles (Figure 2B,C). The required increase of lysosomes for Cd sequestration was apparently achieved by an enlargement of the lysosomal volume in relation to the cytoplasmic space in digestive cells, as expressed quantitatively by the rising volume density of digestive cell lysosomes in Cd-exposed winkles (Figure 5A). This increased loading capacity of digestive cell lysosomes seems to be compromised by a decrease in the lysosomal membrane stability, as shown for *Littorina littorea* exposed to environmental stressors and polyaromatic hydrocarbons [43,63,65].

Overall, there are actually two relevant pools of Cd sequestration in the midgut gland of *Littorina littorea*: apart from the lysosomal compartment, the second pool of Cd sequestration is due to the binding of the metal to cytosolic MT, which in the present study is documented by the Cd-induced increase of the MT mRNA concentration (Figure 3). It is important to discover how these two metal pools (the cytosolic MT-related and the lysosomal one) interact. The data of the present study show that both pools react to the Cd load in a timely response between the 3rd and the 7th day after the start of Cd exposure (compare Figure 3B and Figure 5A). Our interpretation is that, at the beginning of Cd exposure, most of the metal may first be sequestered by constitutive and de novo synthesized MT. At this stage of Cd accumulation, the MT-associated Cd pool may prevail. Due to ongoing MT turnover and degradation [36], however, a certain part of the liberated Cd ions would be bound to de novo synthesized MT, whereas a minor proportion of the metal (perhaps still attached to half-degraded and denatured MT) would end up in digestive cell lysosomes for final storage. From there, some Cd would leak out back into the cytoplasmic space due to lysosomal membrane destabilization (see above). Eventually, impaired Cd-loaded lysosomes would be discarded by fecal excretion, probably together with extruded digestive cells. The viability of this hypothesis was confirmed by studies in mussels, where metal-loaded lysosomes and cell debris were detected in the lumen of midgut gland tubules [24,35,74].

The increasing presence of lipofuscin granules in the digestive cells of Cd-exposed winkles (see Figure 6) may be the consequence of enhanced cellular proliferation and re-structuration due to Cd exposure. This probably leads to an increment of autophagy and cell turnover, accompanied by impaired digestion within the lysosomal compartment. In other gastropod species too, Cd apparently stimulates the proliferation of endoplasmic reticulum and midgut gland tubular cells, followed by a concentration-dependent increase in the formation of residual bodies and lipofuscin granules [72].

#### 3.2.2. Basophilic Cells

The results observed at the histological level show that some cellular alterations induced by Cd exposure in the present study developed faster than expected [33]. This holds particularly for the increase of the volume density of basophilic cells (Vv_BAS_) in the midgut gland tubules of Cd-exposed periwinkles. This was, in the present study, the fastest responsive parameter among all observed variables, with significantly increased values only 12 h after the start of Cd treatment (Figure 7A). As a direct consequence of this increase, the tubular epithelium of the midgut gland undergoes a process of cell type replacement, which is considered to be a general biomarker for environmental stress in bivalves and gastropods [40,44,73,75,76]. Under control conditions, the majority of the midgut gland tubular epithelium is composed by digestive cells. Upon stress exposure, however, the proportion of digestive cells drops down, resulting in a gradual increase of the number of basophilic cells, which eventually may constitute the majority of the midgut gland tubular epithelium (see above). During the period of recovery, Vv_BAS_ levels remained significantly above control values, even though a slight (but statistically insignificant) tendency towards normalization could be observed [32]. In fact, one may expect that, in the course of a longer period of recovery, the original number of digestive cells may gradually be restored in the absence of Cd, giving also rise to a normalization of the Vv_BAS_ values [32]. The fact that periwinkles were forced to stay underwater for the entire course of Cd exposure could have caused, in the present study, an incomplete restoration of control values during the observed time span of recovery compared to previous studies [24,32,45].

#### 3.2.3. Connective Tissue Calcium Cells

During Cd exposure, the number of connective tissue calcium cells increased slightly in response to Cd exposure, particularly in the CdH group, but returned to control levels after the period of recovery (Figure 7B). Connective tissue calcium cells are constituents of the mantle, foot, albumen gland and visceral complex (including the midgut gland) of gastropods and are closely related with blood vessels. They contain calcium carbonate, which is thought to regulate the calcium content, contributing to the ionic balance and the maintenance of haemolymph pH [77]. Metals such as Cd, Cu and Sr can also be incorporated into the mineral granules inside the connective tissue calcium cells [35]. Thus, in the present study, the slight increase of connective tissue calcium cell density in Cd-exposed winkles (Figure 7B and Figure 9) is probably related to the presence of Cd in these cells. On the other hand, their calcium carbonate stores must remain disposable to rapid mobilization upon metabolic demand [77]. This would probably also lead to a rapid liberation of the trapped Cd^2+^ ions which, because of their similar ionic radii, may be confused with Ca^2+^ ions by cellular transport mechanisms [78] and Ca-dependent signalling pathways [68]. Hence, the inactivation of Cd^2+^ on behalf of calcium may be one of the important protective roles exerted by MT in the midgut gland tissue of *Littorina littorea*.

#### 3.2.4. Cd-Induced Loss of Tissue Structure and Integrity

Clearly, Cd-induced alterations of cellular structure and integrity, as well as cell type-specific replacements, must have an impact on the appearance and integrity of the organs and tissues constituted by the respective cell types. For example, the structural degradation of digestive cells and their gradual replacement by basophilic cells in the midgut gland tubular epithelium of Cd-exposed winkles is apparently reflected by a loss of integrity at the tissue level. This can be quantified, as in the present study, by changes of the connective tissue to digestive tubule (CTD) ratio (see Figure 10A), or by alterations of the lumen visibility in midgut gland tubules (see Figure 10B). At least the first of these parameters—the CDT ratio—shows an obvious degradation in dependence of Cd exposure in both the CdL and CdH-treated winkles (Figure 10A). Similar results were reported from other studies in different mollusc species exposed to environmental stressors [79,80].

At the same time, as tissue alterations appeared in the midgut gland, a decrease of tissue integrity was also assessed in the epithelia of the digestive tract (Figure 11), which became clearly evident in histological preparations (Figure 12). As in the case of integrity loss in the tubular epithelium of the midgut gland, the decrease of the integrity of epithelial cells in the digestive tract of Cd-treated winkles was, in part, due to epithelial cells losing their height by discharging their apical ends under Cd exposure [39]. This is probably a reaction from the tissues to get rid of their Cd-loaded cells upon exposure [33]. The loss of digestive cells can lead to an impairment of digestion capability.

#### 3.2.5. The Biomarker Potential of Assessed Parameters

Due to their short-termed capacity for being induced at sub-lethal concentrations of Cd, many of the parameters assessed in the present study may have a strong potential for their application as biomarkers in environmental monitoring [81]. This holds particularly for cellular biomarkers assessed in different midgut gland cell types and for sub-cellular biomarkers such as lysosomal impairment, most of which show a great responsiveness and rapidity in their patterns of reaction.

The midgut gland of molluscs is the crossroads for metabolic regulation, participating in the mechanisms of immune defense and the homeostatic regulation of the internal medium, as well as in the processes of stress response and metal detoxification. In fact, cellular endpoints such as changes in the lysosomal structure and lysosomal metal accumulation have previously been used with success to assess the effect of pollutants on molluscs [22,82].

The start of the MT mRNA increase depends, among other reasons, on the Cd concentration applied during exposure, showing that MT mRNA concentrations start to rise earlier at higher Cd concentrations in the CdH group compared to a somewhat delayed reaction in the CdL group (Figure 3B). In contrast, there was no clear relationship between Cd concentration of the exposure medium (CdL or CdH) or Cd concentration factors in the midgut gland of periwinkles on the one hand, and the values of transcriptional induction of the *MT* gene on the other (see Figure 4). This resembles previous findings reporting that the level of expressed MT protein in the midgut gland of Cd-exposed winkles was hardly affected by the intensity of Cd exposure [55], compromising to some extent the applicability of MT upregulation as a biomarker for Cd stress in *Littorina littorea*.

**Figure 13:**
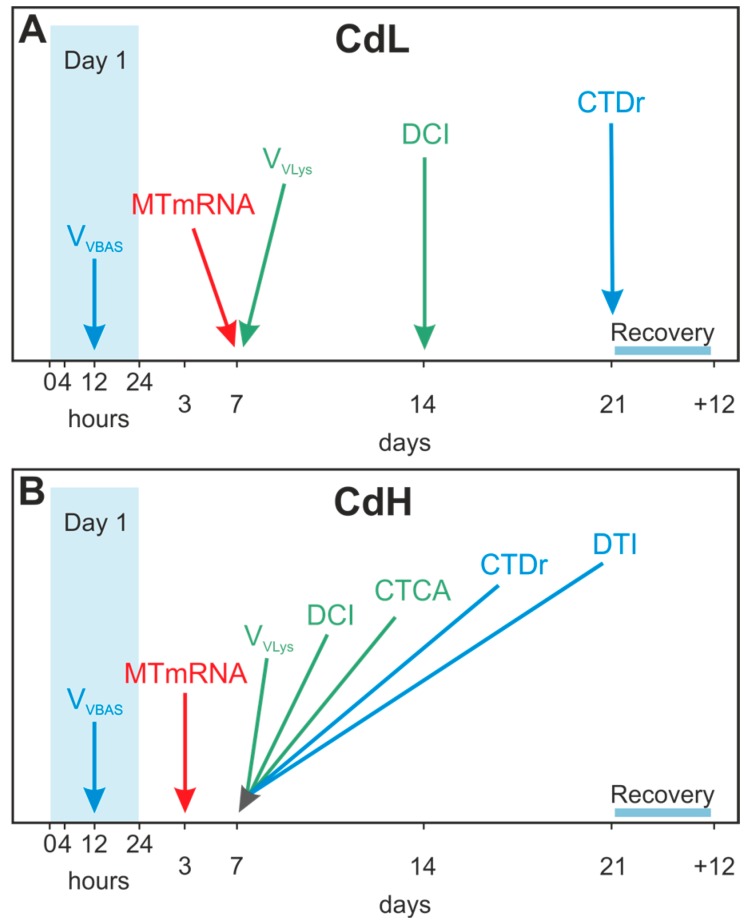
Time course of the onset of molecular (red), cellular (green) and tissue-specific alterations (blue) in *Littorina littorea* exposed to sub-lethal low (CdL) (**A**) and high (CdH) (**B**) Cd concentrations through 21 days of exposure, followed by a 12 days period of recovery. Abbreviations: MT mRNA: MT mRNA copy numbers, Vv_LYS_: Volume Density of lysosomes in digestive cells of midgut gland tubules, DCI: Digestive cell integrity of midgut gland tubules; CTCA: presence of connective tissue calcium cells in the midgut gland, V_VBAS_: Volume density of basophilic cells in the epithelium of midgut gland tubules, DTI: Digestive tract integrity. CTDr: Connective tissue to digestive tubule ratio.

Taken together, the time patterns of the different molecular, cellular and tissue-specific response reactions to Cd exposure in *Littorina littorea* may very well provide a valuable biomarker battery approach for the purposes of biomonitoring (Figure 13). It appeared that the timeframe for the onset of the different response reactions became narrower at higher Cd concentrations (CdH) compared to the lower exposure (CdL). In this time-dependent succession, the first parameter which showed distinct reactions at both Cd concentrations (CdL and CdH) was, interestingly, the volume density of basophilic cells (V_VBAS_) in the periwinkle’s midgut gland, with significantly increased values after only 12 h of exposure (see Figure 13). This indicates that Cd-stressed periwinkles respond to the intake of the metal by starting the re-modelling of their cellular composition of the tubular tissue in the midgut gland at an early stage. The increase of the MT mRNA concentration follows next, prior to or together with the activation of the lysosomal compartment (Figure 13), suggesting that cytosolic inactivation of Cd by binding to MT and lysosomal Cd accumulation may be complementary processes of detoxification that go hand in hand, as explained above. All other cellular and tissue-specific response reactions to Cd exposure followed afterward.

In spite of their general validity as biomarkers, many of the cellular and molecular variables assessed in the present study may also respond to environmental stimuli not directly related to Cd stress. This has been shown, for example, for increasing MT mRNA concentrations in periwinkles exposed to anoxia and freezing [31], or for alterations of the lysosomal compartment in midgut gland digestive cells due to seasonal fluctuations or pollution impacts in the intertidal habitat of periwinkles. In the present study, too, the volume density of the lysosomal system (Vv_LYS_) in the first three days of exposure must be more related to the experimental design than to the effects of Cd exposure (see Figure 5A), as suggested by the concomitant alteration of this variable in all exposure groups, independent of Cd concentration. A possible reason for this may be the fact that, in the present study, periwinkles were forced to remain immersed under water for the entire study. This condition may have given rise to alterations of the metabolism of periwinkles, with possible impacts on their digestive cycles, as previously reported for subtidal mussels [83]. Evidently, confounding factors like these must be observed when applying cellular and molecular variables of *Littorina littorea* for purposes of environmental monitoring.

## 4. Materials and Methods

### 4.1. Experimental Set-Up

*Littorina littorea* collected in Scrabster (Scotland) were purchased from a commercial dealer (Arrainko SL, Mercabilbao, Bilbao, Spain). Specimens with a maximum height of between 20–30 mm were selected and acclimated to experimentation conditions in a seawater flow through the system for a week before experimentation (Salinity: 33‰; Temperature: 17 °C). A group of 175 individuals was stated as the control group. Another two groups of 175 individuals each were subjected to nominal Cd concentrations (applied as CdCl_2_) of 0.25 mg·Cd/L (“Cd Low”, CdL) and to 1 mg·Cd/L (“Cd High”, CdH) for 21 days, respectively. Real Cd concentrations measured in the seawater were 0.035 ± 0.005 mg/L for control conditions, 0.243 ± 0.006 mg/L for CdL exposure, and 0.852 ± 0.021 mg/L for CdH exposure (means ± standard deviations, *n* = 9). pH values in the seawater throughout the exposure were 7.72 ± 0.18 for control conditions, 7.76 ± 0.24 for CdL exposure, and 7.79 ± 0.21 for CdH exposure (means ± standard deviations, *n* = 27). The O_2_ concentration in the seawater was constantly at ~ 4 mg/L.

During the experimentation period, winkles were maintained in 40 L of naturally sand-filtered well-seawater from a clean place (Plentzia, Bizkaia, Spain) and fed *ad libitum* with *Bifurcaria bifurcata.* Seawater and food were changed every second day, and a net was installed in the interface air-water of each tank to maintain periwinkles always underwater.

Winkles were sampled after 0, 4, 12 h and 1, 3, 7, 14 and 21 days of exposure. At the end of the exposure period, winkles were maintained for recovery in clean seawater for 12 days without Cd supply. 10 winkles per experimental group (controls, CdL and CdH) were sacrificed performed at each exposure time and after the recovery period. The midgut gland/gonad complex was dissected out and processed as described below to obtain the different endpoints.

### 4.2. mRNA Isolation, Allelic Variant Screening and Quantitative Real-Time PCR of the Reference Gene

Three Cd-exposed individuals were used for PCR and sequencing in order to confirm the primary protein structure of the reference MT of *Littorina littorea* (see below). An additional 20 individuals were used for the screening of allelic MT variants (see below).

For each experimental time point, four individuals of *Littorina littorea* were dissected on an ice-cooled stainless steel plate and total RNA was isolated from ~10 mg of homogenized (Precellys, Bertin Instruments, Montigny-le-Breonneux, France) hepatopancreatic tissue with the RNeasy^®^Plant Mini Kit (Qiagen, Venlo, The Netherlands) applying on-column DNase 1 digestion (Qiagen). RNA was screened for integrity visually on an agarose gel and quantified with the RiboGreen^®^RNA Quantification Kit from Molecular Probes (Invitrogen, Karlsruhe, Germany) on a VICTOR™X4 2030 Multilabel Reader (PerkinElmer, Waltham, MA, USA). First, strand cDNA was synthesized from 450 ng of total RNA with the Superscript^®^ IV Reverse Transcriptase synthesis kit (Invitrogen, Life Technologies, Waltham, MA, USA) in a 20 µL approach for subsequent Real-time Detection PCR. The remaining tissue was processed further for Cd analysis as described below.

The primary structure of the *Littorina littorea* MT (GenBank Acc.Nr.: AY034179.1) [31] was confirmed by PCR and sequencing (*n* = 3). In a screening for allelic variation by sequencing 20 PCR—amplified and cloned individuals of *Littorina littorea*, 5 distinct isoforms (*n* = 3) were detected. PCR primers located in the untranslated region (sense 5′-CTGACGAGTGAACTGTTTTT-3′ and antisense 5′-GATGGGGAATGAGAAAATG-3′) were applied. The respective sequences were submitted to GenBank and are accessible under the acc. nrs.: KY963497 (allelic variant 1), KY963498 (allelic variant 2), KY963499 (allelic variant 3), KY963500 (allelic variant 4) and KY963501 (allelic variant 5).

Quantitative Real-time Detection PCR of the *Littorina littorea* MT was performed on a Quant studio 3 (Applied Biosystems, Thermo Fisher Scientific, Waltham, MA, USA) using Power SYBR Green (Applied Biosystems, Thermo Fisher Scientific, Waltham, MA, USA). The transcript with the defined amplicon length of 84 bp was amplified using the following concentrations and primers: *Littorina littorea* litt. sense, 900 nM; 5′-AATACGGAGCGGGTTGCA-3′ and *Litoorina littorea*. antisense, 900 nM; 5′-AGCGACAGTCCTCCTTACAGTTG-3′ applying the following protocol of 40 cycles: denaturation at 95 °C for 15 s, annealing and extension combined at 60 °C for 60 s. The 10 µL PCR reaction contained 1 µL of cDNA and 1× Power SYBR Green PCR master mix, 1× U-BSA and sense and antisense primer. Primers were designed using the Primer Express 3.0 software (Applied Biosystems) and optimal primer concentrations were assessed with a primer-matrix followed by dissociation curves. Calibration curves from amplicons were generated to determine C_q_ values (PCR efficiency ~92%) for copy number analysis using the Thermo Fisher Cloud Software, Version 1.0 (Life Technologies Corporation, Carlsbad, CA, USA).

### 4.3. Metal Analysis

Cd concentrations in the midgut gland tissues and seawater were assessed by flame atomic absorption spectrophotometry. After oven-drying the dissected midgut gland at 65 °C, the samples were pressure-digested in 2 mL tubes (Eppendorf, Hamburg, Germany) with a 1:1 mixture of nitric acid (Suprapure, Merck, Darmstadt, Germany) and deionized water in an aluminum oven covered with a heated lid at 69 °C until a clear solution was obtained. All samples were diluted to 2 mL with deionized water and Cd concentrations measured by graphite furnace atomic absorption spectrophotometry (model Z-8200, Hitachi, Tokyo, Japan). Standard metal solutions in 1% nitric acid were used for calibration. Accuracy of metal measurements of the midgut gland was verified with certified standard reference material (TORT-2, Lobster Hepatopancreas Reference Material for Trace Metals; National Research Council Canada).

### 4.4. Sample Processing for Microscopy

The midgut gland/gonad complex of control and exposed winkles was fixed in neutralized phosphate buffer with formaldehyde at 4%, dehydrated in a series of ethanol baths and paraffin, embedded using a Leica ASP3005 tissue processor and sectioned at 5 µm with a Leica RM2125RTS microtome for histopathological analysis (Section 2.3.3) and autometallographical staining (Section 2.3.4). Another portion of the midgut gland/gonad complex was dissected out, frozen in liquid nitrogen and stored at −80 °C, then sectioned at 8µm with a CM3050s Leica cryotome for histochemical analysis.

### 4.5. Histopathology

Paraffin sections (5 µm) were stained with haematoxylin-eosin (H/E) in order to analyze the integrity of the midgut gland, gonad and gills.

The volume density of basophilic cells (Vv_BAS_) and the connective tissue area per digestive diverticula area ratio (CTD) were quantified by means of stereology as an indication of whether changes in cell-type composition and in the amount connective tissue occurred or not [24]. Counts were made in three randomly selected fields in one midgut gland slide per winkle (six winkles per sample). Slides were viewed at 40× objective (final magnification ~400×) using a drawing tube attached to a light microscope. A simplified version of the Weibel graticule multipurpose test system M-168 [83] was used, and hits on basophilic cells (b), digestive cells (d), diverticular lumens (l) and interstitial connective tissue (c) were recorded. Vv_BAS_ was calculated according to the Delesse’s principle [83], as V_VBAS_ = VBAS/VEP, where VBAS is the volume of basophilic cells and VEP the volume of midgut gland epithelium. The CTD ratio was calculated as CTD = c / (b + d + l). 

The general condition of the midgut gland was systematically analyzed under an Olympus BX61 microscope following a semi-quantitative approach. Briefly, the integrity of each organ was ranked with a value ranging from 0 to 4, where the highest (control) integrity was 0 and the highest possible damage was 4. The clear visualization of the limits of the lumen of the midgut gland tubule was ranked from 0–4, where 0 meant the impossibility of properly seeing the lumen under light microscope and 4 meant that the lumen was totally visible and their limits defined. The same rank (0–4) was used for semi-quantitative assessment of the presence of calcium cells in the interstitial connective tissue. The criteria used for the semi quantification of the histopathological endpoints are explained in Table 1.

### 4.6. Autometallography

The intra-lysosomal accumulation of metals was determined in paraffin-embedded sections following the autometallography procedure of Danscher [51]. Paraffin sections (5 µm) were dewaxed in xylene and hydrated in decreasing ethanol degree baths, once hydrated slides were left in an oven at 37 °C overnight. Tissue sections were covered using temperate and homogenized commercial silver enhancement kit (BBI Solutions) solution (initiator and enhancer solution mixed in a 1:1 ratio) under safety light conditions following the product instructions. Sections were developed for 20 min and then washed for 2 min with tap water. The slides were mounted using Kaiser’s glycerin gelatin. Metals were visualized as black silver deposits (BSD) using an Olympus light microscope. The quantification of the BSD extent (Volume density of BSD; Vv_BSD_) by image analysis could not be performed because the size of the deposits was in many cases under the detection limit of the system.

### 4.7. Stereology of Digestive Cell Lysosomes

The histochemical activity of β-glucuronidase was demonstrated in unfixed cryotome sections as in [84]. Sections (8 µm) were cut in a CM3050 cryotome at a cabinet temperature of −25 °C, collected into warm glass slides and stored at −40 °C until required for staining. Sections were tempered at room temperature for 5 min and then transferred to the substrate incubation medium consisting of 22.4 mg of naphthol AS-BI-β-d-glucuronide dissolved in 0.96 mL sodium bicarbonate (50 mM) and made up to 80 mL with 0.1M acetate buffer (pH 4.5) containing 2.5% NaCl and 12 g of polyvinyl at a 20% (*w*/*v*) concentration as colloid stabilizer. Sections were incubated for 20 min at 37 °C in a water bath with constant agitation. Then slides were rinsed in 2.5% NaCl at 37 °C for 2 min and stained at room temperature for 10 min and under darkness conditions, with 1 mg/mL fast garnet GBC in 0.1 M phosphate buffer (pH 7.4) plus 2.5% NaCl. Sections were then fixed in Baker’s calcium formol (4% formaldehyde, 1% calcium chloride, 2.5% sodium chloride) for 10 min at 4 °C and rinsed in distilled water. Finally, sections were gently washed in distilled water and mounted in Kaiser’s gelatine.

A stereological procedure was applied to quantify the structure of the digestive cell lysosomes in periwinkles, using an image analysis system. The system consists of a B&W-CCD video camera, a Leitz Laborlux light microscope, a computer with video board and BMS software. An objective lens of 100× magnification was used. Binary images segregating lysosomes from digestive cell cytoplasm were obtained by the segmentation procedure, which was manually adjusted in the first measurement of a given section to correct slight differences in staining intensity between different sections. With the image analysis system, the lysosomal volume density (Vv_LYS_ = VL/VC) was generated, where V = volume, L = lysosomes and C = digestive cell cytoplasm. Five measurements were made per midgut gland. The stereological formulae included a correction factor for particles with an average diameter smaller than the section thickness [85]. Sample size was determined based on previous analyses of mean and standard deviation values of the four parameters, which at least resulted in a maintained constant for a sampling area over 16,000 µm^2^ [86]. Since the total area of digestive cells scanned in each measurement was approximately 4000 µm^2^, 5 measurements were made on one single section (total sampling area per mussel 20,000 µm^2^).

### 4.8 Lipofuscin Determination

Lipofuscins were detected using the Schmorl method [87]. Lipofuscins are residual pigments stored in lysosomes, organelles in which the first detectable alterations caused by pollutants can be detected before any other effect on physiological parameters can be observed [88]. Sections (8 µm) were cut in a CM3050S Leica cryotome at a cabinet temperature of −25 °C, collected into warm glass slides and stored at −40 °C until required for staining. First, slides were fixed in fixative as described above for 15 min at 4 °C. After rinsing the sections in distilled water, there were immersed in the reaction medium containing 1% ferric chloride and 1% potassium ferricyanide in a ratio of 3:1 for 5 min. Then, the sections were rinsed in 1% acetic acid for 1 min. At last, sections were rinsed in distilled water and mounted with Kaiser’s glycerin gelatin. The appearance of lipofuscins as bluish granular concretions was analyzed under Olympus light microscope.

### 4.9. Biometry

Biometric measurements were performed with periwinkles from D0 and D21 (Control, CdL and CdH). Whole animal and flesh weights were recorded; and in addition, the maximum length and width of the shells were measured with a caliper up to the nearest millimeter.

### 4.10. Statistics

The statistical analysis was carried out with the aid of the SPSS/PC+ statistical package V.22 (SPSS Inc., Microsoft Co.). For the lysosomal volume density, for the basophilic cell stereology and for the connective to diverticula ratio, one-way ANOVA and a subsequent Duncan’s Posthoc test for multiple comparisons between pairs of mean values was applied (*p* < 0.05). For the semi-quantitative results obtained in the general histopathology, non-parametric Kruskal–Wallis tests were carried out comparing the variances between experimental groups (*p* < 0.05).

Data of qRT-PCR and metal analysis were statistically evaluated by Sigma Plot 12.5. For normal-distributed data, the *t*-test was applied whereas for data failing equal distribution the Holm-Sidak method was used. Statistical significance was set at *p* ≤ 0.05. Additionally, an analysis of variance (ANOVA) was applied to test for significance of time-dependent variations of data (*p* ≤ 0.001).

## 5. Conclusions

1. The exposure of periwinkles (*Littorina littorea*) to sub-lethal Cd concentrations (Cd Low, 0.25 and Cd High, 1 mg Cd/L) over 21 days provoked the successive induction of molecular, cellular and tissue-specific response reactions in the midgut gland and digestive tract of metal-exposed winkles. The assessed parameters were: increase of MT mRNA concentration, volume density of digestive cell lysosomes and lipofuscin formation in the midgut gland tubular epithelium, tubular digestive cell integrity, volume density of tubular basophilic cells, presence of connective tissue calcium cells, as well as tissue integrity of the midgut gland/gonad complex and of the digestive tract.
2. The beginning of alterations of the assessed parameters followed distinct concentration-dependent and time-dependent patterns, where the timeframe for the onset of the different response reactions became narrower at higher Cd concentrations (CdH) compared to lower exposure concentrations (CdL).
3. Interestingly, the first parameter that showed distinct reactions at both Cd concentrations (CdL and CdH) was the volume density of basophilic cells (V_VBAS_) in the periwinkle’s midgut gland, with significantly increased values after only 12 h of exposure. This proves that Cd-stressed periwinkles respond to the intake of the metal by a very early re-modelling of the cellular composition in the tubular tissue of the midgut gland.
4. The increase of the MT mRNA concentration follows next, prior to or together with the activation of the lysosomal compartment of tubular digestive cells, suggesting that cytosolic inactivation of Cd by binding to MT and lysosomal Cd accumulation may be complementary processes of detoxification which interfere with each other. At the beginning of Cd exposure, most of the metal may first be sequestered by constitutive and de novo synthesized MT. At this stage of Cd accumulation, the MT-associated Cd pool may prevail. Due to ongoing MT turnover and degradation (Bebianno and Langston 1998), however, a certain part of liberated Cd ions would be bound to de novo synthesized MT, whereas a minor proportion of the metal (perhaps still attached to half-degraded and denatured MT) would end up for final storage in digestive cell lysosomes. Overall, the near concomitance of increasing MT mRNA transcription with enormous alterations at the cellular and tissue-specific levels reinforces the impression that the increasing MT mRNA concentrations in the midgut gland of periwinkles apparently serve the purpose of protecting *Littorina littorea* from potentially adverse effects induced by Cd^2+^ ions entering the cells or leaking out from impaired cellular structures.
5. An important response strategy regarding Cd stress in *Littorina littorea* is, apart from Cd sequestration by MT and lysosomes, the re-modelling of midgut gland tubules by cell replacement, where digestive cells die at a faster rate than they proliferate, implicating an increase of the number and volume density of basophilic cells, which prevail in tubules of Cd-stressed individuals. In addition to this, there is also a Cd-induced increase of the number of connective tissue calcium cells.
6. The specific Cd-induced molecular and cellular alterations in metal-stressed *Littorina littorea* may themselves be applied as biomarkers in environmental monitoring. Taken together, the time patterns of the different molecular, cellular and tissue-specific response reactions to Cd exposure in periwinkles may well provide a valuable biomarker battery approach.

## Figures and Tables

**Table 1:** Criteria for the semi-quantification of the histopathological alterations observed in the midgut gland of *Littorina littorea* upon Cd exposure: Digestive cell integrity loss, Integrity loss of digestive tract epithelium, Lumen visibility of midgut gland tubules, Presence of Ca cells in midgut gland connective tissue. The appraisal of the apparent histopathological status is ranked in arbitrary units from 0 to 4, with 0 being the control value and 4 the worst possible status.

Endpoint	Score				
	0	1	2	3	4
Digestive cell integrity loss	Control integrity	Slightly lower cells	Lower cells slightly vacuolated epithelium	Low cells Vacuolated epithelium	Disintegrated tissue
Integrity loss of digestive tract epithelium	Control integrity	Slightly lower cells	Slightly lower cells slightly vacuolated epithelium	Slightly lower cells vacuolated epithelium	Disintegrated tissue
Lumen visibility of midgut gland tubules	<10% of the area of the tubule	<30% of the area of the tubule	<40% of the area of the tubule	<50% of the area of the tubule	>50% of the area of the tubule
Presence of Ca cells in the midgut gland connective tissue	<30% of the connective tissue	<45% of the connective tissue	<60% of the connective tissue	<85% of the connective tissue	>85% of the connective tissue
